# Activities of the Right Temporo-Parieto-Occipital Junction Reflect Spatial Hearing Ability in Cochlear Implant Users

**DOI:** 10.3389/fnins.2021.613101

**Published:** 2021-03-12

**Authors:** Eusebia Schäfer, Ana-Elena Vedoveli, Giulia Righetti, Philipp Gamerdinger, Marlies Knipper, Anke Tropitzsch, Hans-Otto Karnath, Christoph Braun, Yiwen Li Hegner

**Affiliations:** ^1^MEG Center, University of Tübingen, Tübingen, Germany; ^2^Department of Otolaryngology, Head and Neck Surgery, Tübingen Hearing Research Centre, University of Tübingen, Tübingen, Germany; ^3^Comprehensive Cochlear Implant Center, ENT Clinic Tübingen, Tübingen University Hospital, Tübingen, Germany; ^4^Center of Neurology, Division of Neuropsychology, Hertie-Institute for Clinical Brain Research, University of Tübingen, Tübingen, Germany; ^5^CIMeC, Center for Mind/Brain Research, University of Trento, Rovereto, Italy; ^6^DiPsCo, Department of Psychology and Cognitive Science, Rovereto, Italy; ^7^Center of Neurology, Department of Neurology and Epileptology, Hertie-Institute for Clinical Brain Research, University of Tübingen, Tübingen, Germany

**Keywords:** EEG, CI, TPO junction, repetition suppression, auditory

## Abstract

Spatial hearing is critical for us not only to orient ourselves in space, but also to follow a conversation with multiple speakers involved in a complex sound environment. The hearing ability of people who suffered from severe sensorineural hearing loss can be restored by cochlear implants (CIs), however, with a large outcome variability. Yet, the causes of the CI performance variability remain incompletely understood. Despite the CI-based restoration of the peripheral auditory input, central auditory processing might still not function fully. Here we developed a multi-modal repetition suppression (MMRS) paradigm that is capable of capturing stimulus property-specific processing, in order to identify the neural correlates of spatial hearing and potential central neural indexes useful for the rehabilitation of sound localization in CI users. To this end, 17 normal hearing and 13 CI participants underwent the MMRS task while their brain activity was recorded with a 256-channel electroencephalography (EEG). The participants were required to discriminate between the probe sound location coming from a horizontal array of loudspeakers. The EEG MMRS response following the probe sound was elicited at various brain regions and at different stages of processing. Interestingly, the more similar this differential MMRS response in the right temporo-parieto-occipital (TPO) junction in CI users was to the normal hearing group, the better was the spatial hearing performance in individual CI users. Based on this finding, we suggest that the differential MMRS response at the right TPO junction could serve as a central neural index for intact or impaired sound localization abilities.

## Introduction

When acting in a multisensory environment, it is predominantly the auditory cues which direct our attention to relevant targets and help us orient in space when the events happen outside the field of view. This skill is of crucial importance for us to become aware of potential pertinent incidents, such as threats. Moreover, spatial hearing enables us to distinguish among different sound sources in complex acoustic environments and to attend only to sounds that interest us. The capacity to follow an auditory stream by virtue of its spatial location is especially important in multi-speaker environments and is referred to as the “cocktail-party effect” (Cherry, 1953). Consequently, impairments and loss of hearing affect orientation in space, attentional control, in addition to communication (Shinn-Cunningham and Best, 2008).

Cochlear implants (CIs) are until now the most successful treatment for patients with severe to profound sensorineural hearing loss as they restore hearing to such an extent that speech recognition is reestablished or considerably improved, enabling verbal communication in numerous deaf and hearing impaired patients (Krueger et al., 2008). Optimizing speech perception in CI users has been the highest priority in the last decades of CI development. However, the rehabilitation of sound spatial localization after cochlear implantation has been comparatively less invested into (Faulkner and Pisoni, 2013), still restricting rare success to gain modifications through automatic gain control of CI devices (Potts et al., 2019).

In normal hearing people, sound source localization in the horizontal plane relies mainly on the comparison of ILD (interaural level differences) and ITD (interaural time difference) cues (Middlebrooks and Green, 1991). Although these sensitive binaural cues are limited or even not available due to unsynchronized auditory input between the ears in CI users (Ausili et al., 2019), some CI users have considerable spatial sound localization performance (Seeber et al., 2004). Monaural spectral cues usually play a much less important role in horizontal sound localization (Macpherson and Middlebrooks, 2002) and these cues are probably absent at the implanted ear. However, adaptive cortical plasticity due to experiences following unilateral hearing loss could make good use of the spectral information (for a recent review, see Kumpik and King, 2019). Spatial hearing in CI users has been studied in bilaterally- (Litovsky et al., 2006; e.g., Brown and Balkany, 2007; Grantham et al., 2007; van Deun et al., 2010; Dorman et al., 2014; Gifford et al., 2014; Choi et al., 2017) as well as in unilaterally implanted users (Vermeire and van de Heyning, 2009; Gartrell et al., 2014; Nawaz et al., 2014; Liu et al., 2016; e.g., Döge et al., 2017). Nevertheless, many of these studies focused on localization performance rather than exploring the neural mechanisms of the central nervous system underlying spatial hearing.

To improve the sound spatial localization performance with a CI, much of the previous work has taken the bottom-up (peripheral) approach concentrating on implant technology as for example improving coding and stimulation strategies (e.g., see Potts et al., 2019). Since some years, however, researchers started to consider the top-down (central) influences in the spatial hearing outcomes in the CI users. Fields that have been investigated under the context of CI imply a contributing role of cortical plasticity and cross-modal interaction (Syka, 2002; Fallon et al., 2008; Moore and Shannon, 2009; Merabet and Pascual-Leone, 2010; Kral and Sharma, 2012; Sandmann et al., 2012; Keating and King, 2013; Sharma et al., 2015; Chen et al., 2017; Stevenson et al., 2017; Stropahl et al., 2017), as well as executive functioning (Kral et al., 2016) to the large variation of CI performance outcome. Indeed, over the first twelve months of implant use, strong learning or accommodation processes with continuous improvements in outcome have been documented (Wilson and Dorman, 2008). These findings point to the importance of cortical plasticity, which enables the brain to use effectively the relatively crude and distorted input provided by the CI. The question is, whether suitable rehabilitation procedures can be established in order to improve sound localization skills in CI users (Tyler et al., 2010; for a case study, see Nawaz et al., 2014)? Any malfunction along the peripheral to the central auditory pathway could cause a deficit in sound localization, assuming that the peripheral auditory inputs were restored via CI (and the ascending auditory pathway was intact as a prerequisite for CI), a central auditory functional assessment would be helpful to reveal or even reestablish the potential of sound localization abilities of a CI user.

As a first step toward a sensitive diagnostic tool identifying the level at which spatial hearing is impaired, our current study aims at investigating the neurophysiological processes of spatial hearing in normal hearing people and CI users at the cortical level. The long-term goal is to develop beneficial rehabilitation programs for hearing-impaired patients using hearing aids or CIs. To this end, we introduce a multi-modal repetition suppression (MMRS) paradigm that is suitable for both testing and eventually training spatial hearing, and that allows to identify brain regions that are involved in the discrimination of sound locations using high-density electroencephalography (EEG). Previous EEG studies have shown the potential of measuring mismatch negativity (MMN) signals to estimate the spectral discrimination abilities of CI users as an objective evaluation tool of their speech perception (Lopez-Valdes et al., 2013; Lopez Valdes et al., 2014). Our approach is based on a combination of repetition suppression or stimulus-specific adaptation (Grill-Spector et al., 2006; Salminen et al., 2015), MMN (Näätänen et al., 2017) and associative learning paradigms. Accordingly, we reasoned that the cortical regions that are sensitive to sound spatial location would display stronger activation when a sound location change occurs after repetitive sound stimulation in the same location. We presented five sound stimuli per trial in a row. The first four identical sound stimuli served as adaptor stimuli and were paired with a flashlight, all at the same location. The light could thus serve as a cross-modal teaching signal when the paradigm is later applied in training sound localization capabilities in hearing-impaired users, by providing concurrent visual information regarding the location of the sound stimulus. In contrast to the four adaptor stimuli, the fifth sound-only stimulus served as a probe that could originate either from the same (SAME condition) or from a deviant azimuth direction (DIFF condition) than the adaptor stimuli. The subject was asked to discriminate the location of the probe sound using a pointing device.

We investigated the neural correlates that exhibit differential responses (MMRS response) to spatially deviant probe sounds (SAME vs. DIFF) as a potential central neural index of intact audio-spatial discrimination abilities in a group of 17 normal hearing participants. The novelty here is using a modified MMN paradigm in the context of impaired sound localization together with advanced cortical source analysis. We hypothesized that this MMRS response elicited by the probe stimulus in the normal hearing subjects, similar to those in previous sound spatial mismatch studies (e.g., Deouell et al., 2006), might be generated beyond the primary auditory cortex, along the auditory “where” pathway (Zündorf et al., 2016), including posterior superior temporal gyrus, i.e., planum temporale (Deouell et al., 2006, 2007; Altmann et al., 2007; Ahveninen et al., 2013; Zündorf et al., 2014), inferior parietal lobule and superior frontal sulcus (Arnott et al., 2004). In particular, stronger fMRI (functional magnetic resonance imaging) responses were observed for sound location changes comparing to sound pattern changes in bilateral inferior parietal lobule, the right temporo-parietal junction and the right anterior insula (Altmann et al., 2007). We localized the MMRS response on the cortex in the normal hearing group to further test whether the MMRS paradigm is suitable of inferring sound localization abilities in 13 postlingual CI users, who exhibit different degrees of sound localization performances. It is hypothesized that the similarity of the spatial temporal patterns of the EEG signals between the CI user and the normal hearing group will indicate a normal function of spatial hearing in our task. We show that reliably measurable brain responses from the MMRS paradigm might be employed as an assessment tool to modulate the top-down influences of spatial hearing rehabilitation in hearing-impaired patients. This is especially important for patients who are unable to indicate the direction of sound sources, for example, due to young age or neurodegenerative diseases.

## Materials and Methods

### Participants

Seventeen normal hearing participants (nine females, mean ± standard age: 24.6 ± 3.8 years, range 19–32 years, all right-handed) took part in the study. Pure tone audiometry was used to verify normal hearing thresholds in the healthy participants. Hearing thresholds in dB HL for the left and the right ear for 10 frequencies between 0.25 and 8 kHz (0.25, 0.5, 0.75, 1, 1.5, 2, 3, 4, 6, and 8 Hz) were measured. Normal hearing was defined as hearing thresholds ≤25 dB HL for each frequency tested.

Additionally, we investigated 13 CI users (three females, 11 unilaterally implanted, two bilaterally implanted, mean age 50.1 ± 15.6 years, range 23–69 years, one left-handed). Their demographic data such as age, the duration of deafness (DOD), the time of CI use, the brand of the implant and the performance of the not implanted ear as well as their task performance of the MMRS paradigm (percent correct responses) are listed in Table 1.

**TABLE 1:** Information about the 13 CI users: age, duration of deafness (DOD) (y), CI use (y), implant type (brand), hearing performance of the not implanted ear, and MMRS task performance (hit rate in percentage).

Age (y)	Side	DOD (y)	CI use (y)	Brand	Not implanted ear	MMRS task (%)
23	R	0.2	1.9	Cochlear	NH	91
29	R	22.4	1.5	MED-EL	NH	–
58	R	2.1	2.3	MED-EL	HI for high frequencies	45
52	R	43.1	1.9	MED-EL	Light HI	54
57	R	24.8	4.4	Cochlear	Light HI	74
28	R	0.3	2.3	Cochlear	NH	68
69	L	0.7	4.4	MED-EL	NH	46
75	L	4.2	4.4	MED-EL	Light HI	34
58	L	0.6	1.6	MED-EL	NH	–
50	L	10–15	0.3	MED-EL	NH	52
51	L	1	3.4	MED-EL	HI for high frequencies	82
57	Bi	*	2.8/1.8	AB/AB	–	38
44	Bi	*	1.8/16.8	AB/AB	–	–

*Severely hearing impaired since birth. AB, Advanced Bionics Corporation (Valencia, CA, United States); Cochlear, Cochlear Corporation (Lane Cove, NSW, Australia); Med-El, Med-El GmbH (Innsbruck, TIR, Austria). NH, normal hearing; HI, hearing impairment.

Normal hearing participants were recruited at the campus of the University of Tübingen. The CI users were recruited through the out-patient clinic at the department of ENT of University Clinics Tübingen (Comprehensive Cochlear Implant Center and Cochlea Rehabilitation Center). All participants reported to have no history of neurological or psychiatric illness. The study was approved by the ethics committee of the Faculty of Medicine Tübingen.

### Apparatus, Stimuli, and Procedure

**FIGURE 1:**
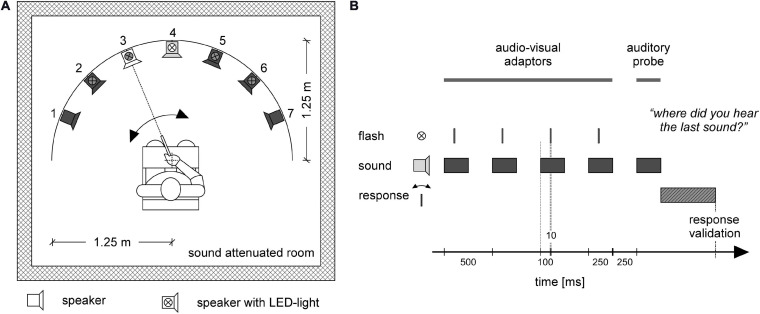
Experimental setup **(A)**, and stimulus and response timing **(B)**. Participants were placed in a sound attenuated room. Seven loudspeakers were placed in front of the participant in a sector covering 135° of a circle with a radius of 1.25 m. The participant was sitting at the center of the circle **(A)**. In each trial **(B)** four audio-visual adaptor stimuli were provided from one loudspeaker together with a flashlight (LED) mounted on top of the loudspeaker. In the depicted example, **(A)** the four adaptor stimuli occur at loudspeaker 4 (loudspeaker in light gray) together with the LED flash presented at the same location (white LED symbol). The series of adaptor stimuli were followed by an auditory-only probe stimulus **(B)** that could occur either at the same position as the adaptor stimuli or at a neighboring location. In the example **(A)** the probe is delivered at position 3 (white loudspeaker). Inactive loudspeakers and LEDs for that trial are depicted in dark gray **(A)**. Participants used a pointing device to indicate the direction of the probe stimulus **(A)**. After moving the pointer in the direction of the sound source, the participant pressed a button on the pointing device to validate their response **(B)**.

The experiment took place in a sound-proof and electrically shielded room. Participants were seated in a chair surrounded by seven loudspeakers all mounted on the same horizontal plane at the approximate height of the participants’ ears, forming a sector of a circle covering 135.0° in steps of 22.5° (Figure 1A). The distance from the center of the subject’s head at ear level to the loudspeakers was 1.25 m. LEDs (light emitting diodes) were installed on top of the five middle loudspeakers at the level of the eyes of the participants. A pointing device for indicating sound directions was attached to the chair at the center directly in front of the participant (for further details about the apparatus, see Zündorf et al., 2011). Since all but one participant were right-handed, they operated the pointer with their right hand (the one left-handed CI user used the left hand). During the experiment, the room was dimly lit so that neither the black loudspeakers nor the LED lights (when off) were barely visible in the darkness. All participants were instructed to fixate a reflective central fixation cross marked at the frontal wall about 10 cm above the central speaker (position 0.0°) during the entire experiment.

**FIGURE 2:**
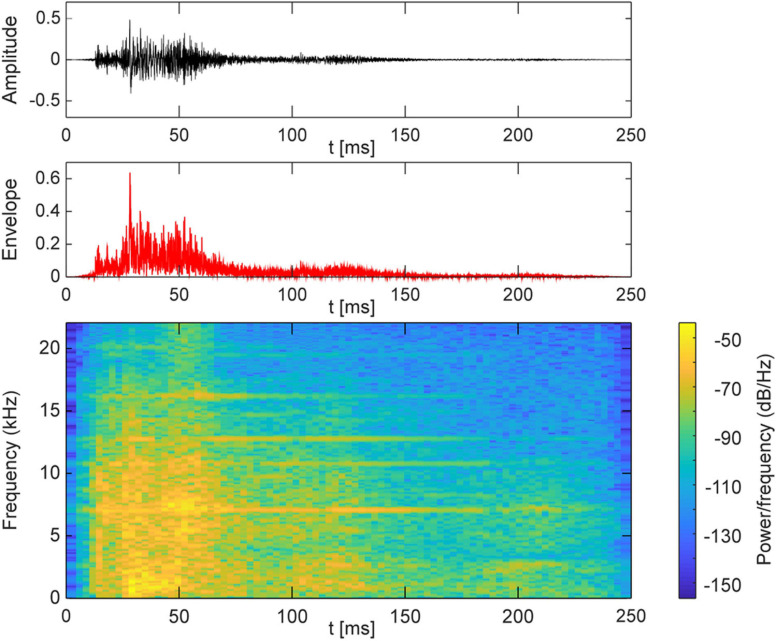
Acoustic characteristics of the sound stimulus.

Auditory stimuli were presented via one out of seven loudspeakers at a time and consisted of a 250-ms broadband frequency signal resembling the sound of a set of keys falling on a solid underground (Figure 2). The loudness of the sound was adjusted to a comfortable level (maximum 50 dB SPL). Correct timing of sound stimuli was assured by using an eight channel USB-audio card (USB soundbox 7.1: Renkforce, Weilheim, Germany). Seven channels of the audio signal were connected to the loudspeakers after amplification. The 8th channel served as trigger channel to control the flashlight and to synchronize the EEG recording with the stimulation. In total there were 750 trials. Each trial consisted of a train of five sounds (Figure 1B), of which the first four sounds came from the same out of the five directions (−45.0, −22.5, 0.0, 22.5, and 45.0°). The inter-stimulus interval was 500 ms. The first four adaptor sounds were accompanied by a light which flashed up for 10 ms. The flashlight was presented 100 ms after the onset of the sound. The last (fifth) sound, i.e., the probe stimulus, originated either from the same azimuth location as the previous adaptor sounds (SAME) or from an adjacent loudspeaker either 22.5° on the left or right (DIFF). The probe sound was not accompanied by a flashlight. The ratio of SAME and DIFF trials was 1:2 (Table 2).

**TABLE 2:** Distribution of stimulus positions and conditions for the 750 trials of the experiment.

	A				B	
Adaptor position	Number	Probe position	Number	Experimental condition	Sound position	Total occurrences
2 (−45.0°)	4 × 150	1	50	DIFF	1 (−67.5°)	50
		2	50	SAME		
		3	50	DIFF	2 (−45.0°)	100
3 (−22.5°)	4 × 150	2	50	DIFF		
		3	50	SAME	3 (−22.5°)	150
		4	50	DIFF		
4 (0.0°)	4 × 150	3	50	DIFF	4 (0.0°)	150
		4	50	SAME		
		5	50	DIFF	5 (22.5°)	150
5 (22.5°)	4 × 150	4	50	DIFF		
		5	50	SAME	6 (45.0°)	100
		6	50	DIFF		
6 (45.0°)	4 × 150	5	50	DIFF	7 (67.5°)	50
		6	50	SAME		
		7	50	DIFF		

The task was to indicate the direction of the probe sound with a pointing device that could be rotated around a vertical axis and that was connected to a rotary potentiometer. By pressing a button on the pointer, subjects signaled the validity of the chosen direction. They were asked to bring the pointer back in every trial such that it was pointing toward the central position. Pointer direction was recorded by the computer controlling the experiment and synchronized offline to the EEG recording.

### EEG-Recording

A 256-channel high-density EEG system was used for the continuous EEG-recording (amplifier NA300 and N400, EGI, Electrical Geodesics, Inc., Eugene, OR, United States). Electrodes were placed in an elastic cap (Hydrogel Geodesic Sensor Net, EGI). Electrode impedances were kept below 10 kΩ. All channels were recorded against a Cz reference. The data was filtered with a highpass of 0.1 Hz and a lowpass of 120 Hz during EEG data acquisition. The sampling rate was 1000 Hz. Participants were asked to fixate at a central frontal cross and avoid any head and eye movements, as well as eye blinks during the measurement. In order to synchronize the stimulation and the recording, the trigger signal was forwarded to the EEG recording system together with the information of stimulation positions.

Electroencephalography-derived cortical source localization results were coregistered to a template head via three fiducial points (nasion, left, and right preauricular points). Fiducials and the individual head shapes of the subjects were digitized using an electromagnetic 3D-digitizer system (Isotrack, Polhemus, Colchester, VT, United States).

### Data Analysis

#### Behavioral Data

To determine the percentage of correct responses for both SAME and DIFF probe conditions, the direction of the pointer with which subjects indicated the perceived direction of the probe stimulus was compared to the position of the loudspeaker which presented the probe stimulus in each trial. The pointing device provided a voltage depending on the pointing angle, a range around each loudspeaker was defined by an individual calibration procedure. In the calibration procedure a voltage range with a lower and upper limit was acquired for each loudspeaker direction. The individual calibration was used in order to rule out any non-linearities and biases of the subject. When the pointer was directed toward a predefined target area around the loudspeaker through which the probe stimulus was presented, the response was counted as correct. If the device was pointing to a region outside the target area the response was counted as incorrect. The extensions of the non-overlapping target areas for all loudspeaker positions were chosen offline for each recording session such that the overall score of correctly identified stimulus directions was maximal.

#### EEG-Data

Electroencephalography-data were analyzed in the MATLAB program (Mathworks Inc., Sherborn, MA, United States) using the open-source Fieldtrip Toolbox (Oostenveld et al., 2011).

#### Preprocessing

Continuously recorded EEG data were first offline highpass filtered at 1 Hz, down sampled to 200 Hz and then lowpass filtered at 35 Hz. The continuous EEG data were segmented into epochs from 0.55 s before the onset of the first sound stimulus until 2.95 s after it. We then applied a Discrete Fourier Transform (DFT) filter on the trial data (epochs) to minimize the first three harmonics of the 50 Hz line noise. Extremely noisy trials due to facial muscle movement were first manually removed by visual inspection. Afterwards an Independent Component Analysis (ICA) was applied to remove heart and eye movement artifacts. In CI users ICA was also used to reduce CI artifacts (Debener et al., 2008). The EEG data were then re-referenced to a common average reference. All trials were baseline corrected with a time window of 0.2 to 0 s prior to the onset of the first sound stimulus in each trial. At the same time, this baseline time window was used to calculate a pre-stimulus baseline covariance matrix needed for noise normalization in the subsequently applied cortical source analysis.

#### Averaging

The segmented, filtered and artifact-cleaned EEG data were averaged for every subject according to the different trial sorting conditions (SAME and DIFF). Trial numbers for conditions to be compared were equalized by randomly selecting as many trials as there were trials in the condition with the least number of trials. Moreover, the trials in SAME and DIFF conditions were balanced not only to the trial number but also to the adaptor and probe sound positions. Error trials (behavior) were not excluded because this might cause very imbalanced trial numbers across conditions in some participants.

#### Source Analysis: Minimum Norm Approach

Averaged responses for SAME and DIFF conditions were subjected to a cortically constrained “minimum norm estimates (MNE)” approach (Hämäläinen and Ilmoniemi, 1994; Lin et al., 2004) for source reconstruction implemented in the Fieldtrip Toolbox package. To this end, individual head models describing volume conduction effects were derived by warping a template head to the individual head shapes. The template head is provided by the “fsaverage” head from the FreeSurfer image analysis suite (Dale et al., 1999, documented and freely available for download online at http://surfer.nmr.mgh.harvard.edu/). The template cortical mesh decimation (ld factor 10 defining the density of the mesh which lead to 1002 vertices per hemisphere) and surface-based alignment was performed with open-source SUMA – AFNI Surface Mapper (Saad and Reynolds, 2012). This standardization process allows further group analysis as the vertex indices of each individual cortical mesh correspond to similar cortical anatomical locations. For more details of this procedure, please refer to the method section of our previous paper (Li Hegner et al., 2018). A boundary element model “dipoli” (Oostendorp and van Oosterom, 1989) was used for the EEG head model with three layers (brain, skull, and scalp with default conductivities of 0.33, 0.0041, and 0.33, correspondingly). Head shapes from individual subjects were manually fitted to the template head shape and individual warping transformation parameters were determined. For each participant, the corresponding warping transformation was applied to the three-layer template head model to create individual head models. Standard EGI electrode positions were fitted manually to the individual head shapes and finally individual leadfields were calculated. The leadfield describes the propagation of activation from each vertex [a cortical source in the head model, 1912 vertices in total excluding the medial corpus callosum area and adding four more regions, i.e., bilateral hippocampus and amygdala, according to the Desikan–Killiany atlas (Desikan et al., 2006) included in the FreeSurfer package] to each EEG sensor, considering the geometrical and electrical properties of the head. Using the MNE approach and applying a common spatial filter for both SAME and DIFF conditions, source power (sum of squares of the three dipole moments, pointing in the *x*, *y*, and *z* directions) at individual mesh vertices were estimated for each time point resulting in a time course of activation power for each vertex and each experimental condition.

#### Source Level Statistics

In a first analysis, the overall EEG source activity was calculated by computing the mean source activity (square root of power, i.e., root sum square of three dipole moments, of each source vertex) across all vertices of the whole cortical mesh for each time point and each condition. In order to obtain the brain activity of all normal hearing subjects the source activity was averaged. A previous EEG auditory azimuth direction deviation study has shown that the mismatch signal peaked within a time window of 100–200 ms after sound stimulus onset (Deouell et al., 2006), in good correspondence with other auditory mismatch studies (for a review, see Näätänen et al., 2017). Thus, in our study the 2100–2400 ms (100–400 ms after probe stimulus onset) time window was taken as a time window of interest for further MMRS analyses on the cortical source level.

In order to resolve the potential differential activation of cortical areas for SAME and DIFF stimulation conditions in time, we divided our time window of interest (2100–2400 ms) in the second analysis into six non-overlapping time windows each of 50 ms duration. Given the vertex neighborhood information, we performed two-tailed dependent-samples *t*-tests comparing SAME and DIFF conditions and used non-parametric cluster-based permutation tests (Maris and Oostenveld, 2007) provided by the Fieldtrip Toolbox to correct for multiple comparison correction in each time window. In short, the mean activities of the two conditions in each time window to be compared were subjected to individual *t*-tests (paired and two-tailed, initial alpha = 0.01, i.e., 0.005 for each tail) for each vertex. Vertices belonging to the medial wall (e.g., Corpus Callosum, indices according to the Desikan–Killiany FreeSurfer atlas) did not enter the statistical analysis. Thus, statistically different neighboring vertices in space with *p*_vertex_ < 0.005 were assigned to a cluster. Each cluster was then characterized by the summed *t*-values across its vertices. The cluster of the largest absolute *t*-value was recorded after each permutation (in total 5000 Monte-Carlo permutations, randomly permuting the assignment of conditions for individual subjects) resulting in an empirical null distribution of the test statistics. As significance criterion a *p*_cluster_ < 0.05 (i.e., *p* < 0.025 for each tail) was chosen. Since six time windows were tested, we used a *p*-value smaller than 0.008 (i.e., 0.05 divided by 6) to control the family-wise error rate for the final cluster-of-interest (COI) selection.

Significant clusters obtained from comparing SAME and DIFF conditions in normal hearing participants were used as COIs for the analysis of the source activities in CI users. While in normal hearing participants, COIs could be defined based on statistical different activations between SAME and DIFF stimuli, this is not very feasible for hearing impaired CI users. Due to their auditory impairment, some of the CI users might not have shown any activation differences between SAME and DIFF conditions. Therefore, the same source analysis procedures as in the normal hearing subjects could not be applied. In contrast, we have chosen a different approach. We calculated the inverse solution for each CI user based on the individual leadfield and the pre-stimulus signal noise. Then separate source activity time courses could be extracted for each COI (derived from the statistical analysis in normal hearing participants) for each CI user.

We reasoned that the cortical differential activation under SAME and DIFF conditions reflected the good performance of the horizontal sound localization task in the normal hearing participants. Therefore, the mean differential time course between SAME and DIFF conditions of the source activity was calculated across 17 normal hearing subjects for each COI. The grand mean differential curve served as COI-specific norm activation to which the differential source activity between SAME and DIFF conditions of individual CI users was compared. To this end, the activity of individual COIs was obtained for each CI user by averaging the source activity across vertices within the corresponding COI. For each COI, samples of the interval ranging from 2100 to 2400 ms of the differential time courses of each individual CI user, and the corresponding norm differential wave of the normal hearing group were subjected to a Pearson’s correlation analysis, resulting in nCI = 10 correlation coefficients per COI. In order to assess whether CI users whose activation patterns strongly deviated from the norm time course would reveal poorer sound localization performance than those CI users with differential wave forms resembling the norm time course, coefficients of individual CI users were entered into a subsequent correlation analysis with individual CI users’ task performance, i.e., total hit rate, as an independent variable. A high correlation value in the analysis thus would indicate the differential COI time course to be a useful predictor for sound localization performance of individual CI users.

## Results

### Normal Hearing Participants

#### Behavioral Data

All normal hearing subjects were confirmed in the pure tone audiometry (hearing thresholds ≤25 dB at the 10 frequencies between 0.25 and 8 kHz). Using the MMRS paradigm subjects were instructed to localize the sound of an auditory stimulus that was preceded by four subsequent and concurrent audiovisual adaptor stimuli originating from the same direction. The fifth probe sound-only stimulus could be either coming from the same direction as the four previous adaptor stimuli or from a position left or right to it. Thus, the chance level for correctly identifying the location of the sound probe is 33.3% assuming that the position of the adaptor stimuli had been perceived.

**FIGURE 3:**
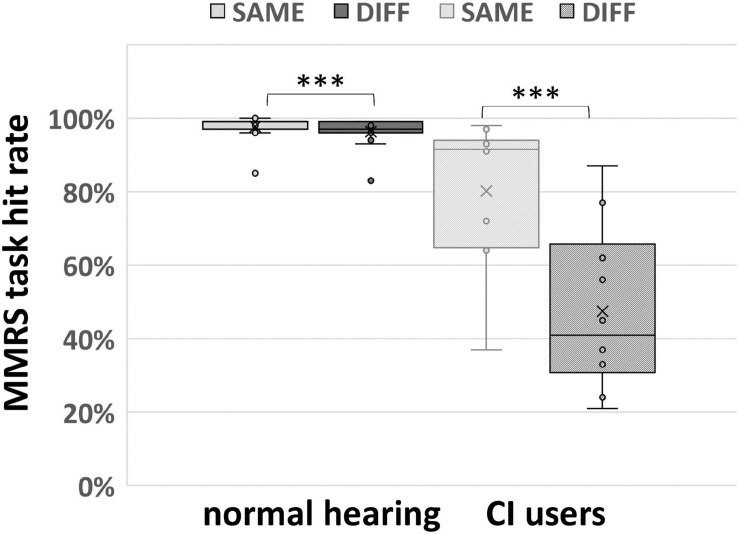
MMRS task performance (separating SAME and DIFF conditions) of 15 normal hearing participants and 10 CI users. The box plots display the minimum, maximum, median, first quartile (25th percentile), and third quartile (75th percentile), respectively. In both normal hearing and CI user groups, participants performed significantly better in SAME comparing DIFF condition (^∗∗∗^ indicates *p* < 0.005, paired and two-sided Wilcoxon signed rank test).

Due to malfunction of the pointing device in two participants, the behavioral data of sound localization of only 15 out of 17 normal hearing subjects could be analyzed. All participants showed very good sound localization performance with ≥94% correct responses except one outlier with a score of 84% (Figure 3). The median and the first and third quartile of the hit rate in the group of normal hearing subjects were (25%-ile / median / 75%-ile) 96.0 / 98.0 / 99.0% and deviated significantly from chance *p* < 0.001 as revealed by a non-parametric sign-test (paired and two-sided Wilcoxon signed rank test). Comparing percent correct responses for conditions SAME und DIFF, another sign-test revealed a significant performance difference (*p* = 0.001) for the SAME condition (25%-ile / median / 75%-ile: 97.0 / 99.0 / 99.0%) than for the DIFF condition (25%-ile / median / 75%-ile: 96.0 / 97.0 / 99.0%).

#### EEG Data

##### Grand average of source activities

**FIGURE 4:**
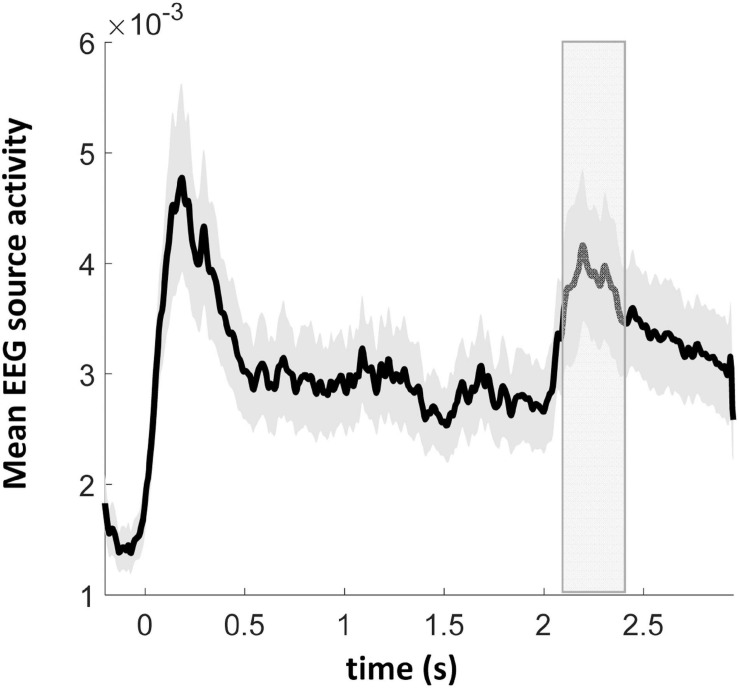
Grand average EEG source activity (arbitrary unit, root sum square of three dipole moments of each source vertex) of 17 normal hearing participants. At the time points of 0, 0.5, 1, and 1.5 s the first four audiovisual adaptor stimuli were presented. At the time point of 2 s the probe sound-only stimulus was presented. The gray rectangle depicts the time window of interest in the cortical source analysis. Gray bounded lines indicate ± SEM.

Electroencephalography overall source activity in the normal hearing participants disclosed a prominent response to the first adaptor stimulus followed by a response decrement for the subsequent three adaptor stimuli. For the probe stimulus, occurring at 2000 ms, its activation revealed a response rebound from the previous activation adaptation (Figure 4). In order to avoid double dipping, we performed SAME vs. DIFF contrast directly on the cortical sources (see below) without performing such a contrast on the mean source time courses, in six subsequent and non-overlapping time windows of 50 ms duration starting from 100 to 400 ms after probe onset, i.e., 2100–2400 ms after trial onset (see the gray rectangle in Figure 4). This time window was chosen on the assumption that the differential signals between SAME and DIFF should be in a similar time window when auditory MMN is elicited, which is peaking at 100–200 ms after stimulus onset (Näätänen and Alho, 1995; Deouell et al., 2006).

Since the probe stimulus was presented without any concurrent visual stimulus (unlike the four previous audiovisual adaptor stimuli), it was further tested whether a dishabituation effect could be demonstrated for the fifth as compared to the fourth stimulus, in the SAME condition only. Paired *t*-tests around the auditory N100 peak (90–110 ms after the corresponding auditory stimulus presentation) and around a secondary peak time window (190–210 ms relative to the auditory onset, since the visual stimulus came 100 ms later than the onset of the auditory stimulus) yielded a significant mean amplitude difference in both the N100 and the secondary time windows. There were significantly stronger activations in the N100 [*p* = 0.006, *t*(16) = −3.15] and the secondary [*p* = 0.0007, *t*(16) = −4.16] time windows during the fifth stimulus (probe with auditory only) in comparison to the fourth stimulus (audiovisual), however, these activations (fifth probe) were significantly weaker in the N100 [*p* = 0.006, *t*(16) = 3.20] and the secondary [*p* = 0.003, *t*(16) = 3.52] time windows comparing to those of the first audiovisual adaptor stimulus. It indicates that dishabituation occurred between the probe stimulus and the fourth audiovisual adaptor stimulus.

##### Cortical localization of the EEG activity

**FIGURE 5:**
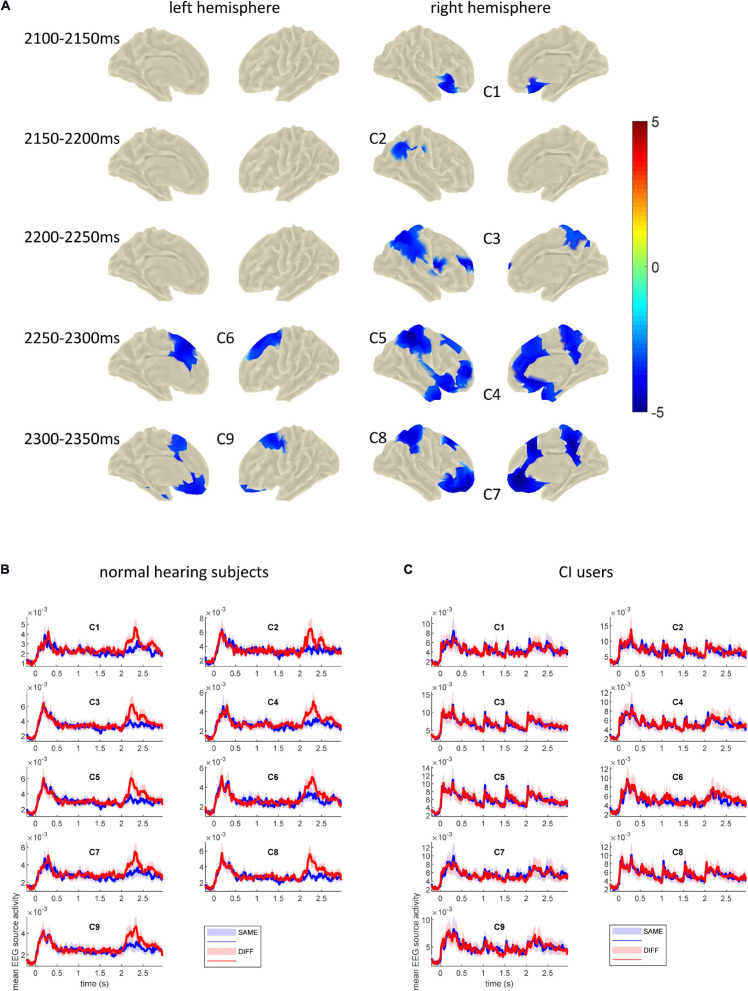
**(A)** Clusters showing significantly stronger activation in DIFF than in SAME conditions across 17 normal hearing subjects tested in the indicated time windows. The onset of the probe sound was at 2000 ms. These clusters were defined as COIs for further analysis in the CI users. Color bar depicts *t*-values. **(B)** Mean EEG source time courses (arbitrary unit, root sum square of three dipole moments of each source vertex) in each COI across 17 normal hearing subjects. **(C)** Mean EEG source time courses in each COI across 13 CI users. The bounded lines indicate ± SEM.

Topographical differences of the cortical activity between SAME and DIFF conditions were assessed in time windows of 50 ms duration from 2100 to 2400 ms with non-overlapping 50 ms steps. Figure 5A shows the time intervals for which clusters of significant differences between SAME and DIFF were found. All clusters revealed stronger activation during DIFF than SAME condition in the time windows tested, except that the last time interval (2350–2400 ms) revealed no significant clusters. All the *p*-values were corrected for multiple comparisons (*p* < 0.05). Corrections were done at the whole brain level for each 50 ms time windows and for the six time windows tested. As shown in detail in Figure 5A, our results revealed that the MMRS effect started from the right inferior frontal cortex / anterior insula (C1, 2100–2150 ms, *p* = 0.002), to the right temporo-parieto-occipital (TPO) junction (C2, 2150–2200 ms, *p* = 0.005), spreading to right parietal (including posterior parietal and precuneus) and superior and inferior frontal areas (C3, 2200–2250 ms, *p* = 0.0002). From then on, the significant MMRS areas became bilateral, while C3 became even more spread in the right parietal (C5, 2250–2300 ms, *p* = 0.001) and frontal areas and to the supplemental motor areas (C4, 2250–2300 ms, *p* = 0.0004), the left premotor and supplemental motor areas also showed up (C6, 2250–2300 ms, *p* = 0.007). In the last significant time window, the right parietal (C8, 2300–2350 ms, *p* = 0.0006) and frontal areas (C7, 2300–2350 ms, *p* = 0.0006) remained similar, the left prefrontal area formed a large cluster together with the left motor and supplemental motor areas (C9, 2300–2350 ms, *p* = 0.001). The mean COI time courses for SAME and DIFF of the normal hearing group and the CI user group are displayed in Figures 5B,C.

### CI Users

#### Behavioral Data

The same MMRS paradigm for sound localization as described for normal hearing subjects was applied in CI users. Due to technical problems, behavioral data of only 10 out of 13 CI users could be analyzed. The performance ranged from 34 to 91%, the median performance was 54.0% with 25 and 75% quantiles of 43.3 and 76.0%. Thereby the sound localization performance to the MMRS paradigm differed greatly between CI users and the normal hearing subjects (who mostly achieved ≥94% correct responses). Comparing percent correct responses for conditions SAME und DIFF (Figure 3), paired two-sided Wilcoxon signed rank test revealed a significant performance difference (*p* = 0.002) between the SAME (25%-ile / median / 75%-ile: 64.8 / 92.0 / 94.0%) and the DIFF condition (25%-ile / median / 75%-ile: 30.8 / 41.0 / 65.8%).

#### EEG Data – Correlational Analysis

**FIGURE 6:**
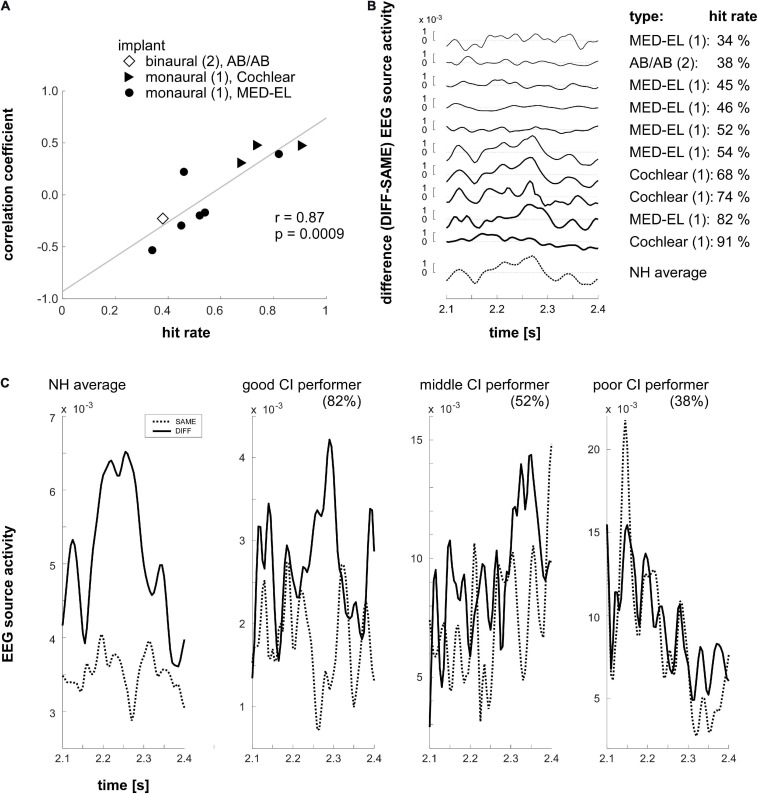
**(A)** Correlation (Pearson, two-tailed) between sound localization performance and the correlation coefficients (Pearson, two-tailed) capturing the similarity of the COI activations between individual CI users and the group of normal hearing individuals. Bilateral and unilateral implantations as well as CI-types are indicated by different symbols: diamond: binaural, AB/AB; triangle: monaural, Cochlear; circle: monaural, MED-EL. For the correlation analysis the COI activation in the time window ranging from 2100 to 2400 ms was considered. A highly significant correlation was found in the right TPO (C2). It indicates that the more similar the individual CI users’ source activity with the activation observed in the normal hearing subjects in the right TPO region, the better sound localization performance had the CI user in the MMRS task. **(B)** Individual differential (DIFF – SAME) EEG source activity of the CI users in the right TPO (C2) region. The depicted source activity is the activity shared with the average source activity of the normal hearing group (NH average; please refer to the main text for further details). Furthermore, hit rates and correlation coefficients of the differential source activity between individual CI users and the mean of the normal hearing group in the 2100–2400 ms time window are shown. **(C)** SAME and DIFF source time courses of the average of the normal hearing (NH) group and three representative CI users with good, middle, and poor sound localization performance (the individual task hit rate is indicated in the brackets).

Clusters differentiating between SAME and DIFF conditions in the normal hearing group were used as COIs for the analysis of source activity of CI users. The differential (DIFF minus SAME) source time courses of each COI were extracted and grand averaged across 17 normal hearing subjects to obtain a “norm” difference curve within the 2100–2400 ms time window. Then such a difference curve was extracted in the same time window in each CI user and was correlated (Pearson’s, two-tailed) with the “norm” curve for every COI. The individual correlation coefficients of the CI users were then correlated with their corresponding sound localization task performance. We found one significant correlation in C2, i.e., the right TPO (*r* = 0.87, *p* = 0.0009, Figure 6A), which suggests that the spatiotemporal profile of the differential MMRS signal is highly relevant to the MMRS sound localization performance of individual CI users. It indicates that the differential activation in the right TPO in the MMRS task could potentially be a cortical neural index and training target of normal sound localization capability. The mean (“norm”) differential curve extracted from the normal hearing group and the differential source activity of the 10 CI users with performance data are shown in Figure 6B. Using principle component analysis (PCA), the time course of source activity common to both, the individual CI users (*a*_*CI*_(*t*) = (*a*_*t*0_,*a*_*t*1_,…,*a*_*tn*_)^*T*^) and the normal hearing control group (*a*_*NH*_(*t*)) was extracted and normalized to the source activity of the NH group. In short, singular value decomposition was applied (*UWV* = *svd*([*a*_*CI*_,*a*_*NH*_])) and the common activity ${\tilde{a}}_{C\,I}$ is computed by keeping only the first component ($\tilde{W}\,\left(1,1\right)=W\,\left(1,1\right)$ and $\tilde{W}\left(2,2\right)=0,and\left[{\tilde{a}}_{C\,I},{\tilde{a}}_{n\,o\,i\,s\,e}\right]=U\tilde{W}V)$. In addition, SAME and DIFF source time courses within the right TPO (C2) of the average normal hearing group, as well as of the three representative CI users with good, middle and poor sound localization performance in our task are shown in Figure 6C.

## Discussion

The present study introduces an MMRS paradigm as an objective non-invasive tool for evaluating human spatial hearing abilities with the goal to establish a robust central neural index for intact audio-spatial information processing. We investigated the neural correlates of horizontal sound localization in 17 normal hearing participants and found that the differential EEG signals (DIFF-SAME, i.e., MMRS responses) upon sound location changes were evident in various cortical regions across time. Accordingly, significant EEG MMRS responses could first be detected in the right inferior frontal cortex, the right TPO junction, and then in the right parietal and precuneus regions, and finally in the bilateral frontal areas. These cortical regions revealed in normal hearing subjects were then taken as COIs when the MMRS paradigm was validated in 10 CI users with highly variable sound localization performance. Interestingly, the similarity of the differential MMRS responses between the individual CI user and the mean of the normal hearing group in the right TPO region was positively correlated to the sound localization performance in CI users. Differential MMRS responses in the right TPO junction could thus be used as a neural index for evaluating and monitoring the success of spatial hearing training in hearing impaired patients and CI users.

### Sound Localization in Normal Hearing Participants and MMRS Response

In the sound localization task, all normal hearing subjects achieved a very good sound localization accuracy that was well above chance level. The excellent performance was expected because angles to be discriminated were 22.5°, which is well above the discrimination threshold (usually below 10° depending on the sound location and frequency) for localizing azimuth sound sources (Mills, 1958; Litovsky and Macmillan, 1994). The rather large angle of 22.5° between sound sources to be discriminated was chosen in our study in order to enable at least some of the CI users to achieve a localization performance better than chance level. Assuming a wide range of performance differences of the CI users we were able to compare the differential MMRS responses, and thus to test the sensitivity of our approach inferring impaired spatial hearing in CI users.

In our analysis, we contrasted evoked potentials for the fifth (probe) stimuli being either deviant or congruent in terms of spatial location from the previous four audiovisual adaptor stimuli. The differential activation for SAME and DIFF probe stimuli, i.e., MMRS response, was hypothesized to be a central neural index for intact spatial hearing. In normal hearing subjects evoked brain responses of the four adaptor audiovisual stimuli showed a strong response magnitude decrease upon every stimulus repetition, indicating response repetition suppression. The fifth auditory-only stimulus deviating from the previous four stimuli with respect to its location (local probability for deviant stimuli: 20 × 66.6% ≈ 13%) elicited a strong discrepancy signal in response magnitude. This rebound signal after repetition suppression or stimulus-specific adaptation might correspond to MMN signal (Näätänen and Alho, 1995; Näätänen et al., 2017), and might be explained under a common predictive coding frame (Carbajal and Malmierca, 2018; Heilbron and Chait, 2018).

Indeed, investigations of the MMN in relation to spatial hearing have been shown to reveal robust MMN responses to changes in sound location with or without auditory attention involved (Paavilainen et al., 1989; Deouell et al., 2006, 2007; Bennemann et al., 2013). The MMRS response investigated in our study may represent an overall mismatch, or expectation suppression (Todorovic and de Lange, 2012), reflecting higher-level analyses of the sound location. It might be related to the MMN or consist of components of the MMN as it occurs in a similar time window (100 ms after stimulus onset) as the MMN signal. However, it might differ from the classical MMN (Näätänen and Alho, 1995) because it involves different brain regions in its generation and propagation.

Interestingly, the activity to the 5th probe stimulus was not only increased with respect to the fourth stimulus in the DIFF condition, but also – even though to a much lesser extent - for the SAME condition. This dishabituation can be explained by the fact that in contrast to the previous stimuli the concurrent flashlight was missing in the fifth (probe) stimulus and thus the adapted audiovisual compound stimuli was followed by an auditory-only stimulus. Furthermore, unlike the preceding stimuli, the probe stimulus was relevant for the task, because it needed to be localized and required the participants’ response. The task relevance of the probe sound might be reflected in an increase of deployed attentional resources leading eventually to an increase in neural activation, not only when adaptor and probe stimuli were different, but also when they were coming from the same direction.

### Neural Correlates Underlying MMRS Response

Sound localization has been shown to be a complex process that involves multiple brain regions (Alain et al., 2001; Clarke et al., 2002; Salminen et al., 2012). Previous neural imaging studies have identified a posterior auditory pathway for sound localization (Arnott et al., 2004; for reviews, see Ahveninen et al., 2014), encompassing the posterior superior temporal gyrus, the planum temporale (Ahveninen et al., 2006; Altmann et al., 2007), posterior parietal and superior frontal areas (Alain et al., 2001). In particular, the right inferior parietal lobule seems to play an important role in updating (Alain et al., 2008) and perceiving sound locations (Zimmer et al., 2006), even when response was not required (Brunetti et al., 2005). The neural correlates underlying the MMRS responses revealed in the current study are largely consistent with these previous findings on brain areas involved in spatial hearing. The MMRS response obtained for probe sound deviating in direction is a compound signal generated from a multitude of brain areas. Neuroelectric source analyses of the EEG activity using a template based minimum-norm approach revealed MMRS signals reflecting various stages of auditory and spatial processing. In detail, significantly stronger activities in the right inferior frontal, right TPO junction, right parietal cortex, right precuneus, bilateral dorsal, medial, and orbital frontal cortices were found in the DIFF comparing to the SAME condition across time. In these areas, brain responses to a change in sound location increased significantly in a time window ranging between 100 and 350 ms after probe stimulus onset.

By analyzing cortical sources contributing to MMRS responses in subsequent time windows, we revealed the spatiotemporal dynamics of the cortical clusters involved in the MMRS task. The right inferior frontal area showing the significant MMRS response in the first time window (100–150 ms after probe stimulus onset) matches well with the view of inferior frontal generator of MMN response possibly in attentional shift (Deouell, 2007). Then the MMRS responses were significant in the right TPO junction spreading to broad posterior parietal region which stayed significant till 350 ms after probe sound onset, as well as the right precuneus. From 200 ms on, first right frontal and then together with the left superior frontal and medial inferior frontal clusters formed a distributed cortical network manifesting significant MMRS responses. In addition to the consensus on the involvement of posterior parietal region in spatial hearing, we found the right precuneus consistently displaying MMRS responses in our sound localization task. The precuneus is part of the posteromedial parietal lobe and is a major hub (Bruner et al., 2017) in various brain functions associated with visuospatial and self-processing operations (Cavanna and Trimble, 2006), as well as auditory spatial computation (Zündorf et al., 2013).

During the later components of the MMRS responses (from 250 ms on), bilateral frontal and right parietal areas are involved in completing the sound localization task. The activation of the supplemental motor areas in medial frontal cortex (Humberstone et al., 1997) can be seen as a correlate of the motoric response of directing the pointer in the perceived direction of the sound and pressing the button. In sum, brain areas contributing to the MMRS responses have been shown to be involved in both, sensory and cognitive processing, but also in the preparation and execution of movements. Thus, MMRS processes appear to be a promising protocol that is capable of disentangling individual steps of the processing continuum spanning from auditory sensation to motor reaction.

### Sound Localization in CI Users

We aimed to find cortical regions which are active during the registration of the sound location change, and relevant for the spatial hearing performance. This is important for the application as a potential evaluation tool for CI users. Sound localization performance was ≥84% in all normal hearing subjects. In contrast, the sound localization performance of 10 CI-users varied within a large range from 34.1 to 91.5% correct responses and reflects the considerable variability of recovered spatial hearing performance in CI users. The CI users presented in the current work are a representative sample for the high variability that is found for spatial hearing after CI implantation. The field of the high variability in performance after the treatment with a CI has to be addressed more extensively in rehabilitation programs and is often described as one of the unsolved “challenges” in implant technology (Faulkner and Pisoni, 2013). CI users who are able to detect sound sources accurately prove that good spatial hearing is possible for CI users despite the disturbed ITD and ILD cues and suggest that an intensive training might be beneficial for improving spatial hearing (Majdak et al., 2013; Firszt et al., 2015; Yu et al., 2018).

Factors that have been shown to affect localization performance in having an effect on neuronal cell physiology and functions in CI users are DOD and duration of implant usage as well as the age at implantation (Cosetti and Waltzman, 2012). The shorter the DOD and the longer a subject had used its implant, the better the localization success. According to the study by Blamey et al. (2013), the duration of CI experience has the largest impact on CI outcome, followed by the age at onset of severe to profound hearing loss, and the DOD and hearing loss. Other factors that have to be considered are variables concerning the device itself (e.g. processing strategy and electrode design), preoperative functions, other disabilities and medical problems, rehabilitation, and auditory training as well as social factors (Cosetti and Waltzman, 2012).

Although in the current study the presented CI users differ widely in their age, background of hearing loss, and the sample number is too small to statistically correlate their performance with biographical, etiological factors, our observed tendencies between performance and factors are in line with the relations previously reported (Blamey et al., 2013). As a first study we chose this inhomogeneous group in order to validate our paradigm in different clinical conditions. Different degrees of deafness and experience with a CI provided sufficient variance in sound localization performance to identify brain regions involved in spatial hearing. The importance of CI experience for good spatial hearing hints to the potential of appropriate training in CI users to restore spatial hearing efficiently via multimodal associative learning and cortical plasticity (Cai et al., 2018). Identifying technical key factors, establishing proper medical treatment and designing effective training strategies for sound localization recovery might help to exploit this potential. Of note, in some CI users the measurement took place after a re-adjustment of the CI, after which the CI users had to get used to the altered auditory perception again. Fitting of the CI, which takes place regularly is an important factor that is needed to be considered when evaluating CI performance. For example, the sound processing and coding strategies might crucially affect spatial hearing performance. Making use of the MMRS paradigm, we suggest to link processing strategies of the CI and sound localization performance in further detailed studies that systematically test sound localization for different frequencies in different environments. Since only a single broad band stimulus was used in the present experiment, we refrain from discussing any relation between the coding strategies of individual products and the performance in the sound localization task. A rigorous study would require a systematic analysis of the effects of different sound types and frequencies on spatial hearing. In a long run, the improved understanding of how the hearing-impaired brain due to distorted auditory input utilizes all the available information and tunes its processing to generate spatial hearing, could help develop intelligent CI processor that integrates “Brain Hearing” technology.

As pointed above, using the MMRS paradigm might enable us to carry out studies investigating a multitude of variables contributing to or impeding the recovery of spatial hearing using CI. In the current study, we are addressing the central processing at the cortical level, thus overriding all the potential factors and only examining the MMRS responses as an end result. MMRS could be a potential neural index to objectively assess the spatial hearing capability of an individual. Once this method is established, future studies could be focused on studying one or few factors mentioned above by recruiting a homogenous group within the range of a certain factor of interest and specific experimental manipulations can be implemented to investigate a particular factor in detail.

### MMRS Response in the Right TPO Correlates With Sound Localization Performance in CI Users

We reasoned that the extent of the similarity (the spatiotemporal profile of the time course) of the differential (DIFF – SAME) activation, i.e., the MMRS response, between individual CI users and the mean of the normal hearing population, could be an indicator of a good sound localization performance of a particular CI user. Indeed, the investigation of the correlation between sound localization performance and a correlation coefficient reflecting the similarity of the MMRS differential curves between individual CI users and the normal hearing group showed that the MMRS response in the right TPO junction could serve as a central neural index for sound localization performance. Our results suggest that the more similar the differential MMRS responses in the right TPO junction between individual CI users and the normal hearing group, the better performance had the CI user. Thus, the spatiotemporal shape of the MMRS response in the right TPO junction could potentially differentiate intact and impaired audio-spatial processing.

The right TPO junction identified from our EEG analysis is in a very similar location to the right inferior parietal area that was revealed as sound localization relevant region in previous positron emission tomography (PET) studies (Bushara et al., 1999; Zatorre et al., 2002), as well as to the right temporo-parietal junction shown in a previous fMRI spatial hearing study (Altmann et al., 2007). From animal electrophysiology and human neural imaging studies, accumulating evidence have moreover pointed to the processing of sound location in a cortical dorsolateral “where” pathway, which originates from the posterior superior temporal gyrus and projects to the parietal and superior frontal area (in contrast to an anteroventral “what” pathway) (Rauschecker and Tian, 2000; Alain et al., 2001; Ahveninen et al., 2006), with possibly some overlap at the inferior frontal region (Zündorf et al., 2016). While contralateral processing is observed in pre-attentive situations (Richter et al., 2009), the right hemisphere dominance in active auditory spatial discrimination tasks (Spierer et al., 2009) is especially reflected in the right posterior parietal cortex (Griffiths et al., 1998; Zatorre et al., 2002; Altmann et al., 2007). In particular, when explicit sound localization was required, Zatorre et al. (2002) have found that the PET activities in the right inferior parietal area could predict the behavioral performance of the subjects. In contrast, the activity in the primary auditory cortex at the superior temporal gyrus did not significantly differ across their different task requirements. Furthermore, the MMRS responses derived from the DIFF/SAME contrast in our study could also be viewed as SWITCH/MAINTAIN auditory spatial attention contrast in two M/EEG studies by Larson and Lee (2013, 2014). They found that the right temporoparietal junction (RTPJ, a very similar region as the right TPO found in our study) was significantly more activated when subjects were asked to switch auditory attention. In particular, the activation of RTPJ was positively correlated with the auditory spatial attention switch performance (Larson and Lee, 2013), and this region is only spatial cue specific (Larson and Lee, 2014). Together with our finding of the behavioral relevance of the right TPO in sound localization performance of the CI users, all the evidence points to a task-specific role of the right inferior parietal cortex in spatial hearing.

Due to the sensitivity and the large amplitude of the MMRS response elicited in our experiment, we are convinced that the MMRS paradigm will be of considerable value for the diagnosis of central sound localization impairments and for outcome monitoring of trainings of spatial hearing in CI users. Specifically, the activity in the right TPO can be potentially used to probe auditory spatial processing in hearing-impaired patients and CI users. Getting a more detailed picture of the areas involved in the processing of audio-spatial information will help to improve the diagnosis of individual deficits in sound localization and could thus be the key for the understanding of the large variability that has been found for the performance in CI users. In this way therapies and rehabilitation programs can be better adapted for individual hearing-impaired patients. Furthermore, training sound localization with additional teaching visual information as it is implemented in the current protocol might help the CI users to strengthen the auditory spatial attention pathway that might have been weakened due to the prior long-lasting deficit in selective auditory attention.

## Data Availability Statement

The raw data supporting the conclusions of this article will be made available by the authors, without undue reservation.

## Ethics Statement

The studies involving human participants were reviewed and approved by the ethics committee of the Faculty of Medicine Tübingen. The patients/participants provided their written informed consent to participate in this study.

## Author Contributions

ES, A-EV, GR, PG, MK, AT, H-OK, CB, and YL: experimental design and reviewing manuscript. ES, A-EV, GR, PG, CB, and YL: data collection. ES, A-EV, GR, CB, and YL: data analysis. ES, CB, and YL: writing manuscript. All authors contributed to the article and approved the submitted version.

## Conflict of Interest

The authors declare that the research was conducted in the absence of any commercial or financial relationships that could be construed as a potential conflict of interest. The reviewer DB declared a past co-authorship with one of the authors MK to the handling editor.
